# Clip-assisted distal traction enables efficient en bloc snare excision of a small rectal neuroendocrine tumor

**DOI:** 10.1055/a-2791-4970

**Published:** 2026-02-17

**Authors:** Zhongshang Sun, Peng Shen, Miaomiao Li, Qi Shi, Yuanyuan Xu, Shijie Ma

**Affiliations:** 1 Department of Gastroenterology, The Affiliated Huaiʼan No. 1 Peopleʼs Hospital of Nanjing Medical University, Huaiʼan, China

A 40-year-old woman underwent screening colonoscopy, which revealed a small rectal subepithelial lesion. The nodule was firm and sessile, without ulceration or central depression. Endoscopic ultrasonography showed a well-circumscribed, homogeneous hypoechoic lesion confined to the second to third echo layers, consistent with origin from the muscularis mucosae and/or submucosa. Given the typical features of a rectal neuroendocrine tumor (NET 1 ) and the small size (≤10 mm), endoscopic en bloc resection was planned to achieve histologically complete (R0) excision while minimizing the risk of muscularis propria injury. Postoperative histopathology confirmed a grade 1 NET.

**Fig. 1:**
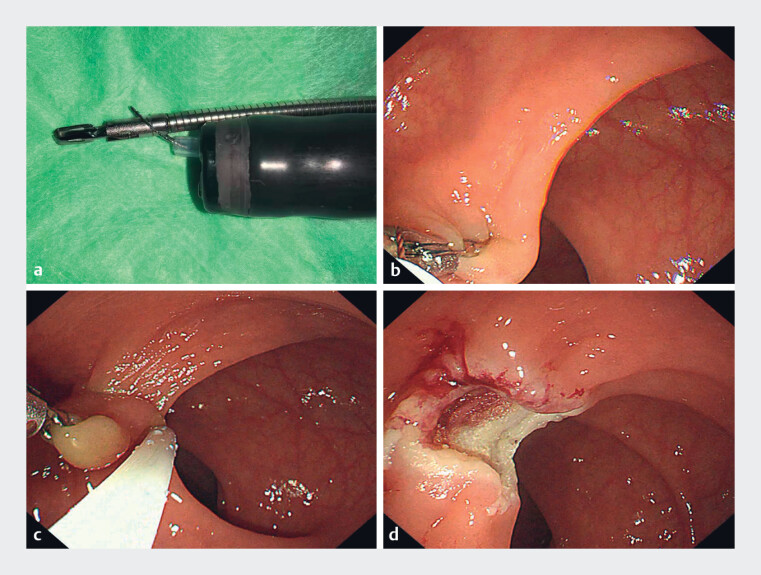
Distal clip traction facilitates safe en bloc snare resection of a small rectal neuroendocrine tumor. **a** A snare was advanced through the working channel and used to secure the clip delivery catheter to the endoscope shaft, enabling stable alignment and controlled clip deployment during subsequent endoscopic manipulation. **b** After the lesion was reached, a metal clip was applied to the nodule apex to provide sustained distal traction, tenting the lesion away from the muscularis propria and enhancing delineation of the resection plane. **c** The snare was positioned at the lesion base to achieve en bloc capture of the entire lesion. **d** No immediate bleeding, deep mural injury, or perforation was observed.

To facilitate efficient and safe removal, we performed a clip-assisted traction-snare resection ( Video 1 ). Before insertion, a snare (JHY-SD-23-230-15-A1, Jiuhong Instrument Co., Ltd, China) with a loop diameter of 15 mm was advanced through the working channel and used to secure the clip delivery catheter to the endoscope shaft, providing stable, controlled deployment ( Fig. 1 **a** ). After reaching the lesion, a metal clip (SD-T-2421-15, Kangjin Instrument Co., Ltd, China) with an opening diameter of 15 mm was applied to the nodule apex and used to apply sustained distal (anal-side) traction, tenting the lesion away from the muscularis propria and improving delineation of the resection plane ( Fig. 1 **b** ). The snare was then positioned at the base to capture the entire lesion ( Fig. 1 **c** ), enabling controlled en bloc resection with intact specimen retrieval ( Fig. 1 **d** ). No immediate bleeding, deep mural injury, or perforation occurred, and the patient recovered uneventfully.

A clip-assisted traction-snare technique for rapid en bloc resection of a small rectal neuroendocrine tumor.Video 1

This technique combines clip-assisted distal traction with snare resection to improve lesion elevation and reduce the likelihood of muscularis propria involvement. By creating a stable traction vector from the lesion apex, it may shorten the procedure time while increasing the probability of en bloc, R0 excision for small rectal subepithelial lesions suspected to be NETs.

Endoscopy_UCTN_Code_CCL_1AD_2AC Endoscopy_UCTN_Code_TTT_1AQ_2AD_3AB
